# Trends in Child Poverty in Sweden: Parental and Child Reports

**DOI:** 10.1007/s12187-015-9337-z

**Published:** 2015-09-23

**Authors:** Carina Mood, Jan O. Jonsson

**Affiliations:** Institute for Futures Studies (IFFS), Stockholm, 11136 Sweden; Swedish Institute for Social Research (SOFI), Stockholm, Sweden; Nuffield College, Oxford University, Oxford, OX1 1NF UK

**Keywords:** Child poverty, Economic problems, Child wellbeing, Poverty indicators, Child reports, Poverty trends, Consequences of income inequality, Poverty in rich countries

## Abstract

We use several family-based indicators of household poverty as well as child-reported economic resources and problems to unravel child poverty trends in Sweden. Our results show that absolute (bread-line) household income poverty, as well as economic deprivation, increased with the recession 1991–96, then reduced and has remained largely unchanged since 2006. Relative income poverty has however increased since the mid-1990s. When we measure child poverty by young people’s own reports, we find few trends between 2000 and 2011. The material conditions appear to have improved and relative poverty has changed very little if at all, contrasting the development of household relative poverty. This contradictory pattern may be a consequence of poor parents distributing relatively more of the household income to their children in times of economic duress, but future studies should scrutinze potentially delayed negative consequences as poor children are lagging behind their non-poor peers. Our methodological conclusion is that although parental and child reports are partly substitutable, they are also complementary, and the simultaneous reporting of different measures is crucial to get a full understanding of trends in child poverty.

## Child Poverty in Sweden

We are constantly reminded by internationally comparative studies that child poverty is widespread also in rich countries (EU 2008; UNICEF 2012). Moreover, there has been a fear during recent decades, following economic recessions and growing income inequality, that child poverty has increased, and some studies have also uncovered trends supporting such worries (e.g., EU 2008; UNICEF 2014).

Child poverty has also become a hot political topic in Sweden, despite low levels of poverty within an international perspective (Bradbury and Jäntti 2001; UNICEF 2012; Eurostat 2009). Trends in child poverty are closely monitored by political parties, authorities and interest organisations (Swedish Social Insurance Agency 2012; Swedish Save our Children 2012; Swedish Ministry of Health and Social Affairs 2004), and the government has identified a set of indicators for following up child policies, including several indicators of economic resources (Swedish Ministry of Health and Social Affairs 2007). The increasing interest in child poverty has come in the wake of the economic depression in Sweden at the beginning of the 1990s, and has been accentuated by reports of growing income inequality since the 1990s (Gustafsson et al. 2007; Jonsson et al. 2010), a trend shared with many Western countries (e.g., OECD 2008; Atkinson 2013).

Studies of trends in child poverty are indispensible for assessing progress and setbacks of welfare states, but defining and measuring child poverty is not a trivial task and differences in concepts and measures may result in different time trends. There are two main issues. The first one applies to poverty research in general, asking whether poverty is best measured in terms of deprivation or in terms of income, and, if the latter, whether a relative or an absolute income measure is preferred. The second issue is whether it is sufficient to measure child poverty in terms of household income or deprivation, or whether we should assess child poverty in terms of children’s own conditions – ideally reported by children themselves.

There is a dearth of studies of trends in child poverty that use several definitions simultaneously. This is unfortunate because such studies can show how robust any trends are, and differences in trends across definitions can give valuable insights into the social forces behind them. Motivated by these advantages, this article aims to study poverty trends in Sweden using both income and deprivation definitions, measured at both the family and the child level, and using reports from both parents, income registers and from children themselves. It is our belief that we are able to improve on earlier studies by this comprehensive approach, and add a more nuanced picture of how children’s economic situation have developed across several macroeconomic changes.

Based on the empirical study of the period 1980/2000–2012, we ask: (i) whether trends are as gloomy as the present political discussion suggests; (ii) whether and how child poverty varies with economic recession and growth; (iii) whether vulnerable groups – in particular children of lone parents and immigrants – bear the burden of increasing poverty, in case such trend could indeed be proven; and (iv) whether household- or child-reported poverty is more strongly associated with non-economic outcomes for children, where we concentrate on here-and-now outcomes, in other words outcomes when children are children. This comparison between household- and child-reported poverty indirectly enables us to evaluate the substitutability or complementarity of parental and child reports on poverty.

Our methodological conclusion is that the simultaneous reporting of different measures is crucial to get a full understanding of trends in child poverty, and any report based on only one indicator or from only one source should be interpreted carefully and regarded as incomplete. In substantive terms, we conclude that the economic situation of child households in Sweden has improved markedly since the beginning of the 2000s, but there are worrying signs that children in families at the bottom of the income distribution are falling further behind the rest. Parents’ connection to the labour market has become more predictive of child poverty, leaving particularly children of lone parents and recent immigrants in a precarious situation. However, although their levels of poverty are very high, their trends are similar to those of other children. Child-reported poverty levels, both in relative and absolute terms, are largely unchanged since the beginning of the 2000s despite trends in parental absolute and relative poverty rates. This is an important insight that could imply intra-household compensation for children in poor families or delayed problems. We conclude that future studies should closely follow the development of relative deprivation experienced by children themselves.

## What is Child Poverty?

According to a definition commonly adhered to, a person is poor who cannot live a life on par with others in the society in which they live (e.g., Sen 1983; Townsend 1979). Thus, poverty is not only a matter of survival – having food, clothes and shelter – but of having the economic means to participate in social life and to meet fellow citizens without shame. Following this lead, child poverty could be defined as a lack of economic resources – stemming from the household’s economy or their own – that prevents children from participating as equals in social life.

Obviously, it is difficult to determine the level of poverty that leads to adverse social outcomes, this being dependent on age, neighbourhood and social network, for example, and it is neither reasonable nor possible to identify local or individual poverty limits. Instead, the normal procedure is to identify the poor indirectly by relating the economic situation to some general idea of “necessary consumption” in a given society at a given time; thus, emanate measures of poverty in terms of income poverty (often categorized into absolute or relative) and measures in terms of material or economic deprivation. At a macro-level, using rates of social assistance (SA)/welfare benefits is also a common indicator of poverty, although open to political tinkering (cutbacks on benefits will register as decreased poverty, for example). Also, receipt of such benefits is not an ideal measure at the household or individual level because the benefits are tailor-made for lifting individuals *out* of poverty.

### Measures of Family Poverty

The most common approach to child poverty defines children as poor if they live in poor families. This is not unreasonable, as the family economy – whether measured in terms of income poverty or deprivation – sets important limits for the quality of housing, the area of residence, and family activities and amenities, and is likely to affect the material conditions and quality of life of all its members. Measures of income poverty classify families as poor if their incomes fall below a pre-determined poverty threshold. In developed countries, this threshold is normally meant to approximate the income necessary for living a life on par with others, a life in “decency”, as Galbraith (1958) put it, and usual practice is to use either a relative or an absolute income poverty threshold.

*Relative income poverty* assumes that that there is a threshold in the income distribution under which living standards are not acceptable (e.g., Townsend 1979; OECD 2008). This is commonly, and rather arbitrarily, set at either 50 % (European Union) or 60 % (OECD) of the median income. Because of the relation to the median income, this indicator captures inequality in the bottom half of the income distribution. An alternative relative measure, less often used, is to define the poor as a given percentile group of the income distribution (but this can obviously not be used for studying trends).

*Absolute income poverty*, sometimes termed “minimum income standard”, sets the threshold at the estimated cost of a given basket of necessities. Because this basket reflects what is seen as acceptable in a given society at a given time, the measure is relative in this time/place sense, and the label “absolute” refers to the assessment of income against a given level of consumption, in contrast to the relative measure’s assessment against other people’s incomes.

Staying closer to the theoretical definition of poverty, one strand of research, rather than taking the indirect route via income, measures living conditions directly (e.g., Townsend 1979; Ringen 1988; Nolan and Whelan 2011; Gordon et al. 2013; Whelan and Maitre 2013). This results in measures of *(economic or material) deprivation* such as subjective measures of economic hardship or more objective information on possessions and cash margin. A branch of this field seeks to integrate the deprivation and income aspects through survey questions on whether families lack some good or activity and, if so, whether this is because they cannot afford it. This is commonly combined with questions on whether the respondent sees the good or activity as a necessity in a given society (Mack and Lansley 1985; Lansley and Mack 2015; Gordon et al. 2013), allowing the estimation of a poverty line based on majority opinions (“socially perceived necessities”), leading to so-called “consensual poverty” estimates saying whether someone lacks a given number of items socially perceived as necessary at a given time and place. An alternative way of combining income and deprivation approaches is to simply define as poor those who fall below both an income poverty line and a deprivation poverty line, so-called “consistent poverty” (e.g., Callan et al. 1993).

### Measures of Child-Level Poverty

Poverty definitions based on the income or deprivation of families assume an equal distribution of resources within a household. This assumption has been criticized from a gender perspective (Millar 2003; Pahl 1990) but is equally questionable from a child perspective. Two children whose parents have equal incomes may themselves have different economic margins and material standards depending on what proportion of the household income they command, and the within-family distribution of economic resources can differ between different types of families. Young people can also have an economy that is partly independent of the parental incomes, for example through their own work for pay, and the access to own money can give a stronger sense of control and freedom than the access to a parent’s money. In order to measure young people’s wellbeing, or level-of-living, direct measures of their total incomes and other economic possessions are therefore necessary (Jonsson and Östberg 2010).

Findings from representative surveys and qualitative interviews suggest that within-family redistribution in families with a strained economy tends to be to the advantage of children, as parents often prioritize children’s needs over their own (Ridge 2002; Main and Bradshaw 2014; Middleton et al. 1997; Gordon et al. 2013). Most of these findings are, however, based on parental reports, either from qualitative accounts or in terms of parental assessments of theirs and their children’s access to necessities. Child-reported data on economic and material resources are scarce but vital, as they give direct information about poverty and deprivation of children in a way that is not filtered through the perceptions of their parents. Letting children convey information about their own situation is also an ethical question, as it is preferable not to let a group’s living conditions be represented by others.

Several qualitative studies use children as informants (for reviews, see Ridge 2011; Redmond 2008a), while few studies use child-informant survey data to construct child-centred poverty measures (for exceptions, see Skevik 2008; Jonsson and Östberg 2010; Main and Bradshaw 2012; Main 2014; Gross-Manos 2015), and even fewer have data that allow for studying trends over time. Data elicited directly from children on several dimensions of economic resources are available for Sweden since 2000 (Jonsson and Östberg 2010), which means that we can now study not only levels but also trends over time in poverty as reported by children themselves.

### Our Measures of Child Poverty

The measures discussed above all have their pros and cons, and our view is that there is no strong theoretical argument to prefer one over another. Using a children’s rights perspective, Redmond (2008b) similarly concludes that none of the dominant theoretical frameworks on poverty suggests a clear-cut definition of child poverty. Many empirical studies also show a relatively small overlap between poverty using different measures (e.g., Mood and Jonsson 2014; Halleröd and Larsson 2008; Whelan et al. 2001), which could be interpreted as poverty being generically multi-faceted, although different definitions of poverty tend to be similarly related to sociodemographic characteristics (Jonsson and Östberg 2004). In light of all this, our preference is to show a comprehensive picture of trends in child poverty, using several indicators and reports from parents, registers and children themselves. This allows us to see how sensitive trends are to different definitions, and any observation of systematic differences in trends across definitions opens up for an increased understanding of what aspects of poverty they capture and how these different aspects vary with other societal changes. Table 1 outlines our course by showing the types of indicators we base our analyses on.

**Table 1:** Types of poverty indicators and informants used

Type of poverty measure	Source of information about poverty	
	Household/parental data	Child-reported data
Absolute	Absolute income poverty; Material/economic deprivation; Social assistance	Pocket money; Income from own work; Material possessions; Cash margin
Relative	Relative income poverty	Ability to afford to keep up with friends

Two of our variables intend to capture the relative deprivation element of poverty by explicitly building comparisons with others into the measure: at the parental level, we use relative income poverty, while on the child level we ask children whether they can afford a social participation and consumption level on par with their friends. We also use a range of measures to capture absolute poverty, by which we mean measures that make no reference to other people’s living standards: at the family level, we use a measure of absolute income poverty (minimum income standard), several indicators of parent-reported deprivation, and one measure of recipiency of social assistance. At the child-level, we use child-reported pocket money, income from work, material possessions, and cash margin. Data sources and variables are presented in sections 3 and 4.

## Data Sources

We draw on several data sources, mostly recent Swedish survey data, complemented with register information on social assistance and income from tax registers. The adult surveys used have sample sizes of around 6000 to 11,000 respondents per wave/year, while the child surveys (for ages 10–18) are around 1000 respondents per wave. The data sources are listed in Table 2, with details in Appendix 1.

**Table 2:** Data sources and poverty indicators from household (hh) and direct child information

Data sources	Years used	Data owner	Poverty indicators
HEK (Hh economy)	1991, 1993–2012	Statistics Sweden	Relative income poverty Absolute income poverty
LNU (Level of living survey)	1981/91/2000/10	Swedish Institute for Social Research	Hh cash margin Hh economic crisis
ULF/EU-SILC (Survey of living conditions)	1980–2007/ 2004–12	Statistics Sweden/ Eurostat	Hh cash margin Hh economic crisis Worry about economy
Child-LNU	2000/2010	Swedish Institute for Social Research	Material possessions Cash margin Income (pocket/pay)
Child-ULF	2001–2010	Statistics Sweden	Relative poverty Material possessions Cash margin Income (pocket/pay)
Register data	1980–2012	Statistics Sweden	Social assistance Household incomes

## Variables

Our analyses focus on the following poverty indicators, which are described briefly here, with more details in Appendix 2:

### Household Poverty (Recalculated to Per-child Units)

- *Absolute poverty* (low/minimum income standard): disposable equivalized income below the poverty threshold, defined as a minimum income/living standard.
- *Relative poverty*: disposable equivalized income below 60 % of the median income.
- *Social assistance*: the household received SA some time during the year.
- *Material/economic deprivation* (or problems) is measured using several different indicators, all from survey questions:
  - Cash margin: the ability of raising a sum of money (around €1500 in today’s value) within a week, if needed;
  - Difficulties to make ends meet/economic crisis; and
  - Worried about the private economy.

### Child Poverty

- *Income*: child allowance/pocket money and work for pay.
- *Material resources/deprivation*: lacking common (mostly individual) possessions.
- *Cash margin*: unable to raise a small sum of money on short notice.
- *Relative poverty*: cannot afford to buy things that friends have (consumption), cannot afford to join friends for events, etc. (participation).

## Trends in Poverty Among Families with Children

### Household Economic Deprivation, 1980–2012

**Fig. 1:**
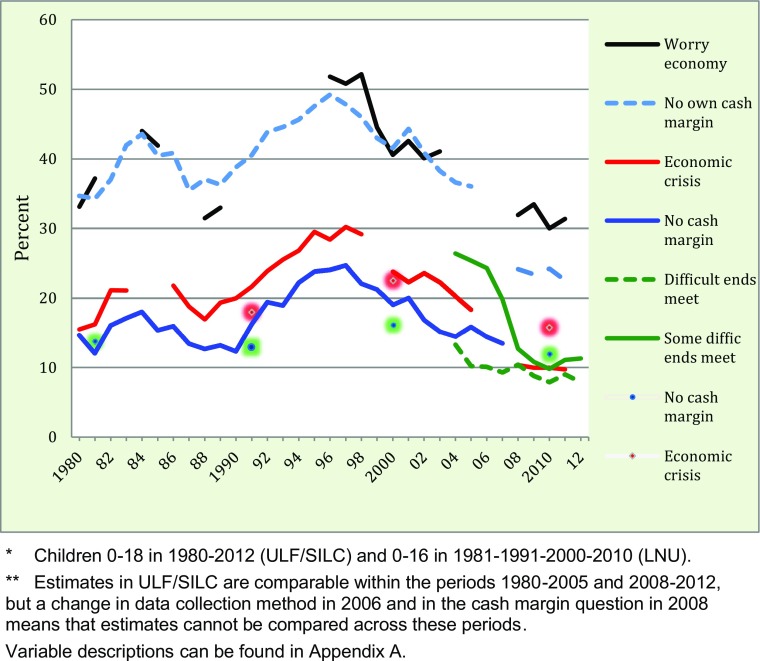
Trends in the percentage of children* living in families with different economic problems, 1980–2012**

In the public debate on child poverty, the focus is often on the most recent annual ups and downs, which certainly can be felt by families living on the margin. However, more important than the temporary (often “accidental” or stochastic) bumps are the longer-term trends. Finding data that can cover long time periods is difficult, but we use two surveys on the basis of which we can study economic deprivation since 1980, although not all indicators are available for the whole period. The annual ULF surveys contribute to the information underlying the curves in Fig. 1, except for those beginning in 2004 or later, which are part of EU-SILC. The LNU survey results are shown as dots for the years the study was conducted (1981, 1991, 2000, and 2010).^1^

Figure 1 shows the development of child poverty over time in terms of the economic deprivation of families with children. The statistics are based on information from parents who respond to questions about the household, but we recalculated the percentages according to the number of children in each family, thus making children the basis of the analysis. The exception is the 2004–2012 estimates of “making ends meet” from EU-SILC, which refer to the proportion of child households.

In the longer time perspective, it is not any specific trend but the fluctuations in economic deprivation that stand out. Such problems were uncommon during the economic boom of the late 1980s but increased rapidly following the economic crash that started in autumn 1991 in Sweden and had repercussions up to 1996–1997. This downturn mirrored what many other countries experienced in 2008–2009. For example, in 1990, 12 % of children (aged 0–18) lived in households with no cash margin, while the corresponding figure for 1997 was 25 %. During this period, the proportion of children living in families who had difficulties meeting their economic needs increased from 20 to 30 %. This was an unprecedented worsening of the economy of families with children.

Following the high poverty rates in 1996–1997, the recovery was slow at first, and it was not until 2005–2006 that these economic problems had returned to pre-recession levels. All in all, poverty rates in terms of deprivation remained unusually high for a good decade. For many children this period represents much of their childhood.

On a positive note, the two surveys support the conclusion that the proportion of children in households with economic troubles has decreased rather steadily since 1996. In the ULF studies, it is unfortunately impossible, due to changes in methods and survey questions, to record comparable data after 2005, but we can analyse trends from 2008 and onwards. From 2004, EU-SILC data can be used. The overriding conclusion is that the recent period, from around 2004 to 2010–2012, is characterized by subsiding rates of economic deprivation in child households. The results from the LNU survey are important here because they add comparability in indicators of cash margin and subjective economic crisis, and they support the conclusion that the proportion of children in households facing economic problems in 2010 is somewhat lower than in 1991, before the recession.^2^

### Changes in Household Income Poverty and Social Assistance

While the trends in economic deprivation can be studied quite far back, it is difficult to find comparable data on income poverty predating 1991, from which year there are reliable data from the annual Household Finances Survey (HEK). We use this survey for studying income poverty and social assistance between 1991 and 2012. Again, measures are at the household level, but we express them as the proportion of poor children.

**Fig. 2:**
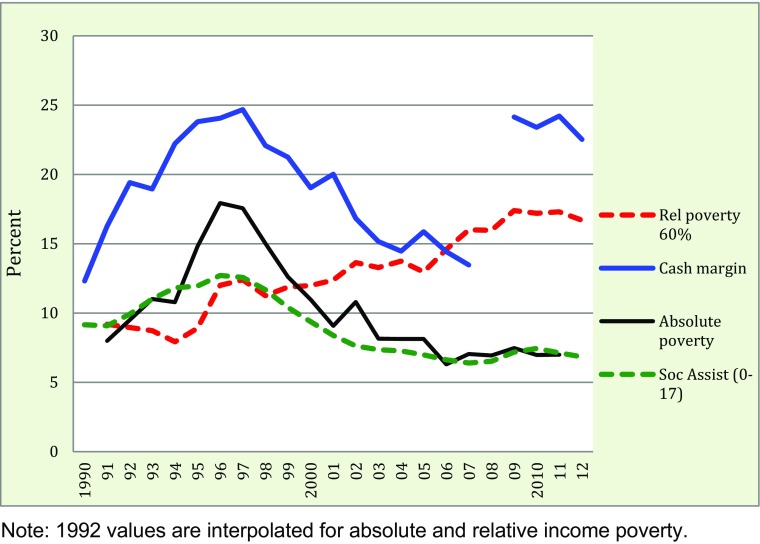
Trends in child poverty according to four indicators, 1990–2012. Proportion of children (0–19) in households (i) with disposable equivalized incomes below the absolute poverty line, (ii) below the relative poverty threshold (EU, 60 %), (iii) lacking cash margin, and (iv) receiving social assistance

Figure 2 shows both the household absolute and relative income poverty, in addition to SA and – taken from Fig. 1 as a benchmark – economic deprivation in the form of lacking cash margin. It is evident that this indicator teams up with absolute income poverty and SA recipiency in following a counter-cyclical pattern: during the recession poverty levels increased, and with the economic improvement following this they decreased. Many families with children suffered economically during the crisis: the proportion of children in families in absolute income poverty (that is below the minimum income standard) increased from 8 to 19 % in a few years. Just as we saw in Fig. 1 for economic deprivation, the period after 2008 has not witnessed much change.

The results above contrast sharply against the trend for relative household poverty rates, which tended to fall during the recession (1991–1994), but showed, overall, increasing figures during the long recuperation. Thus, relative income poverty has been, for most of the period we study, pro-cyclical: decreasing in bad times and increasing in good. This is not a very good quality of an indicator of poverty, as it lacks face validity.^3^ However, it is still an interesting measure, we believe, in conjunction with statistics on absolute income poverty. The reduction of child poverty in the 1996–2006 period in Sweden was due to rising real incomes in child households, but from 2006 real incomes stalled for those at the lower part of the income distribution at the same time as those higher up could maintain some growth. The result was a noteworthy increase in relative but no change in absolute income poverty, reflecting the important fact that children in poorer homes slid further behind other children without being “compensated” by improving purchasing power. One can hypothesize that the relative dimension becomes all the more important in a stagnating economy, but whether the increasing income inequality trickles down to children is an empirical question which we turn to when studying children’s own economy.

While we find it natural to relate poverty trends to economic up- and downturns in a descriptive sense, it is obvious that the causal story behind is more intricate and far beyond the scope of this analysis. Apart from macroeconomic factors, child poverty trends are dependent on, at the least, demographic factors (e.g., immigration, divorce rates, fertility) and policy changes (e.g., family, social, and tax policy). In Fig. 2, the pattern of increase in relative income poverty since 2006 in Sweden, for example, is likely be a result of a rolling tax reform that had as its primary goal to reduce tax on employment at the expense of benefits of various kinds (the main source of income for non-employed). During this period, relative household poverty increased from 60 to 90 % for children with non-employed parent(s) – in contrast, it was merely 3 % for children with two employed parents (Mood and Jonsson 2014).

Because non-employment is such a major, and growing, source of child poverty in Sweden, the dependence on market rewards for the disposable household income is critical. A consequence is that children of lone parents and immigrants are particularly vulnerable groups in Sweden just as in most studied countries (Gornick and Jäntti 2011; Smeeding et al. 2009; Eurostat 2015). Closer inspection of our data verifies that the levels of poverty are much higher for these groups; but the trends are quite similar to other groups’. Children in these at-risk categories are exceptionally sensitive to economic up- and downturns: absolute income poverty affected every third child of lone parents and every second child of immigrants in 1996, dropping to half of these discouraging figures in 2010. While the improvement was great, both the level of and amplitude in poverty rates make these groups a prime concern for policy.

### Recent Child Poverty Trends – an International Perspective

Our results in Figs. 1 and 2 are interesting in an international macroeconomic perspective: they demonstrate how little the 2008–2009 recession hit Swedish children, especially as compared with the recession in the 1990s. The Swedish economy was restructured following the depression in the early 1990s, and although there was a temporary drop in gross domestic product (GDP) in 2008–2009, economic recovery was swift and left almost no traces in economic deprivation or income poverty. In comparison, in some other European nations, such as Ireland, Iceland and Greece, the increase in child poverty rates was substantial (UNICEF 2014).

In difference to the most recent international recession, the one in the 1990s had both sudden and long-term negative effects on child poverty in Sweden. If this experience is anything to go by, it might take a whole decade for child poverty in the economically most affected countries in 2008–2009 to return to pre-recession levels.

## Poverty Among Children

When measuring poverty directly among children, the method must be partly different from the study of families with children. It is difficult to know which economic resources children command because only a small minority of them earn their own incomes, and it is impossible for them (and probably for their parents) to estimate how large a proportion of the household income goes to them. Some get regular (weekly or monthly) allowances from their parents, or they receive the child allowance, which is a universal benefit in Sweden of around €110 per month and child. Some instead get money when they need, some work regularly, while others hardly have any cash at all. Even if it is important to measure children’s economy directly, it is not possible to do so with great precision. We will therefore use several complementary measures. Each has some weakness, but we believe that together they give insights about trends in children’s economic precarity that would not be possible without reports directly from children themselves.

### Relative Poverty: Consumption and Participation

We begin with indicators of relative poverty among children. Questions about whether a responding young person can join their friends in taking part in events (participation) and whether they can afford to buy things that their friends have (consumption) both relate to the economic situation of children’s most tangible reference group. These indicators thus capture the social dimension of poverty.

**Fig. 3:**
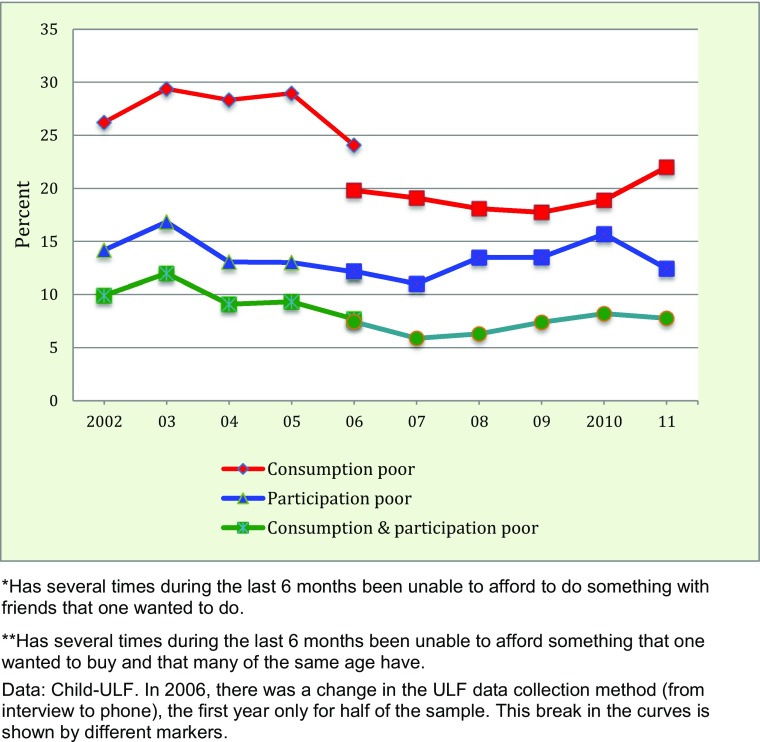
Trends in the percentage of children aged 10–18 who often have problems with participation*, consumption**, and both participation and consumption, 2002–2011

Comparable indicators are available from Child-ULF 2002–11, studying children aged 10–18, and Fig. 3 shows that 8–12 % in this group experienced economic problems with both participation and consumption.^4^ Between 2003 and 2007, this proportion decreased to 6 %, but has since increased somewhat. The trends for participation and consumption diverged between 2007 and 2011, but the sample was quite small in 2011, making the estimates for the end of the period less reliable.

The take-home message from Fig. 3 is that children’s relative poverty fluctuated during the 2000s but without any discernable trend. During the same time, as we noted in Fig. 2, the relative income poverty among families with children increased rapidly.^5^ The theory behind the relative poverty measure says that when low-income earners are amassed at levels far away from the median income earners, their problems with living a life on par with others will grow. The fact that this has not happened for youths is therefore notable, and raises the question why the trends are not aligned.

There are at least five possible answers. First, parents (and perhaps grandparents) may compensate their children economically, so when the family economy falls behind they allocate relatively more resources to their children. Secondly, the social consequences of increasing poverty may be muted because children’s aspiration levels follow their dwindling resources. Thirdly, if the reference group for children is not the “median kids” but their equally poor schoolmates or neighbours, residential and school segregation may be a mitigating factor. Fourthly, the validity of the relative income poverty measure may be wanting, as the experience of poverty may simply be picked up better with a measure reflecting purchasing power. Fifthly, there is a risk for a lag in the social consequences of increased relative poverty, meaning that it is vital to follow child outcomes for some time after an upturn in relative income poverty rates.

### Material and Economic Deprivation

Another important aspect of children’s economy is material possessions, though notoriously difficult to measure, in particular as we have no information on brands or prices, just products (e.g., a mobile phone rather than an iPhone). We choose to show the possessions by item, which helps in understanding the trends, as the necessity, price and preference for different items change over time, and not necessarily at the same pace. Because our data covers only a few items we caution against interpreting them as indicators of a complete set of material possessions.^6^

**Fig. 4:**
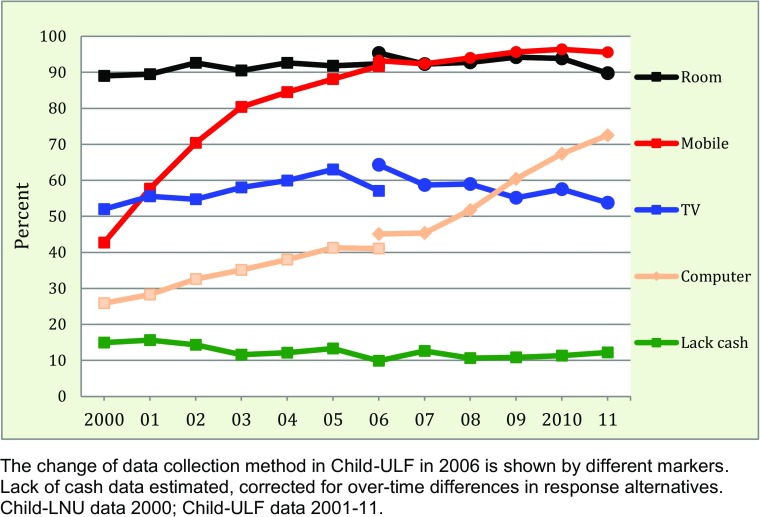
Trends in child-reported material standard, 2000–2011. Proportion of children aged 10–18 who have their own room, mobile phone, TV, or computer; and who lack cash margin

Previous research has established that Swedish children enjoy a high material wellbeing in an international perspective (UNICEF 2012; Bradshaw and Richardson 2009), and there is no sign of deterioration during the period we study (Fig. 4). Around 90 % of 10–18-year-olds have their own room, a proportion that has been constant over time. More than half have their own TV, and the technical development is reflected in the fact that the possession of a mobile phone and personal computer has grown tremendously.^7^

An indicator of economic deprivation that we used at the household level is cash margin, which is also available as a child-reported measure based on a question of whether the child can raise a sum of money (around €10) on short notice if necessary. Slightly more than 10 % lack this possibility, a proportion that naturally is highly age-dependent (not shown). However, this proportion is more or less stable over time.

Access to an own room and a private TV is fairly constant over time, but there is an enormous increase in the proportion of those having their own mobile phone or their own computer. Even if conclusions must be tentative, this is in line with the growth of real incomes during the period, with a concomitant decrease of absolute poverty, but it also reflects the increasing use of and perceived need for mobile phones and computers in everyday life.

### Children’s Access to Own Money

Not having access to own money is an important aspect of poverty, especially for children old enough to be out on their own, without parents. Recurrent incomes most often come from parents, but older children also work during weekends, in the summer, or when school is out. For our trend analysis, we draw on questions to children about their incomes, divided into pocket money/allowances and own work income. It is difficult to know how well we cover their total net income, both because young people acquire money from other sources (e.g., grandparents) and because we do not know whether they also have to cover costs (e.g., sharing their work income with parents). Neither can we assess to which extent children who lack financial resources instead get material resources (something which would reduce their deprivation but still not be equivalent to money in terms of freedom of action). However, while conclusions about levels of income are prone to such measurement problems, the study of trends in incomes should be more reliable as measurement problems are unlikely to vary much over time.

**Fig. 5:**
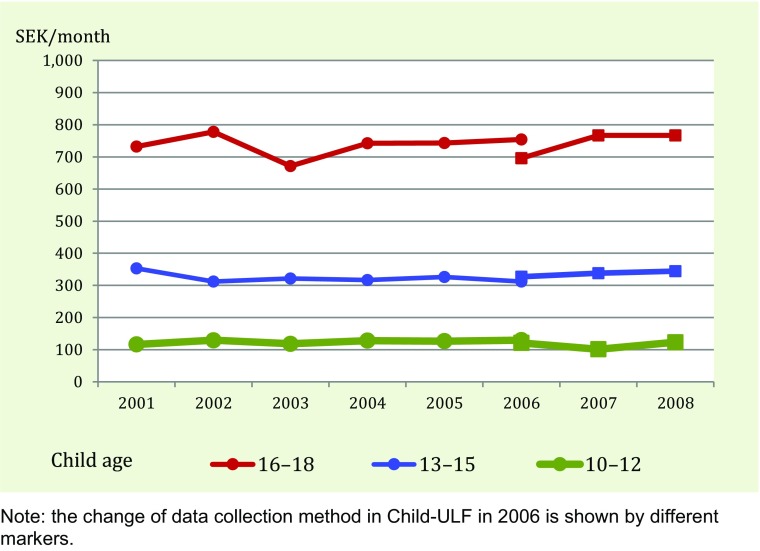
Trends in children’s incomes from allowances/pocket money, 2001–2008. Average sum per age group. SEK per month

More than 80 % of 10–18-year-olds have regular incomes from their parents, a proportion that has been more or less constant during the period 2001 to 2008 (the last year the question was asked). The average sum was also relatively constant, around €40 per month. In the category without regular incomes, most claim that they get money from their parents when needed (an “on-demand economy”), but we were not able to ascertain how much money this entails. Both the regularity and size of the incomes from parents are strongly dependent on the child’s age. The small group (3–5 %) that report that they never get any money from their parents, for example, is dominated by younger children. Figure 5 also reveals that while the level of income is age-specific, there is no change over time in either age group. The oldest (16–18 years old) receive around €80 per month and the youngest (10–12 years old) around €11 throughout the period.

Getting money from extra work is an alternative for older children.^8^ Around 16 % of 16–18-year-olds work every week during school terms, 13 % some time during the month, and 70 % hardly work at all (results not shown). It is more common to work during summer breaks – around half of all 16–18-year-olds have done so during the most recent break. Important for our purposes here, this extent of extra work for pay has not changed during the period 2001–2011.

### Poverty Among Children: Vulnerable Groups

In the analyses of household poverty, we identified as particularly vulnerable groups children residing with only one parent and children of immigrants. Is this pattern reflected when measuring poverty directly at the child level? The answer is yes, but not unambiguously so.

**Fig. 6:**
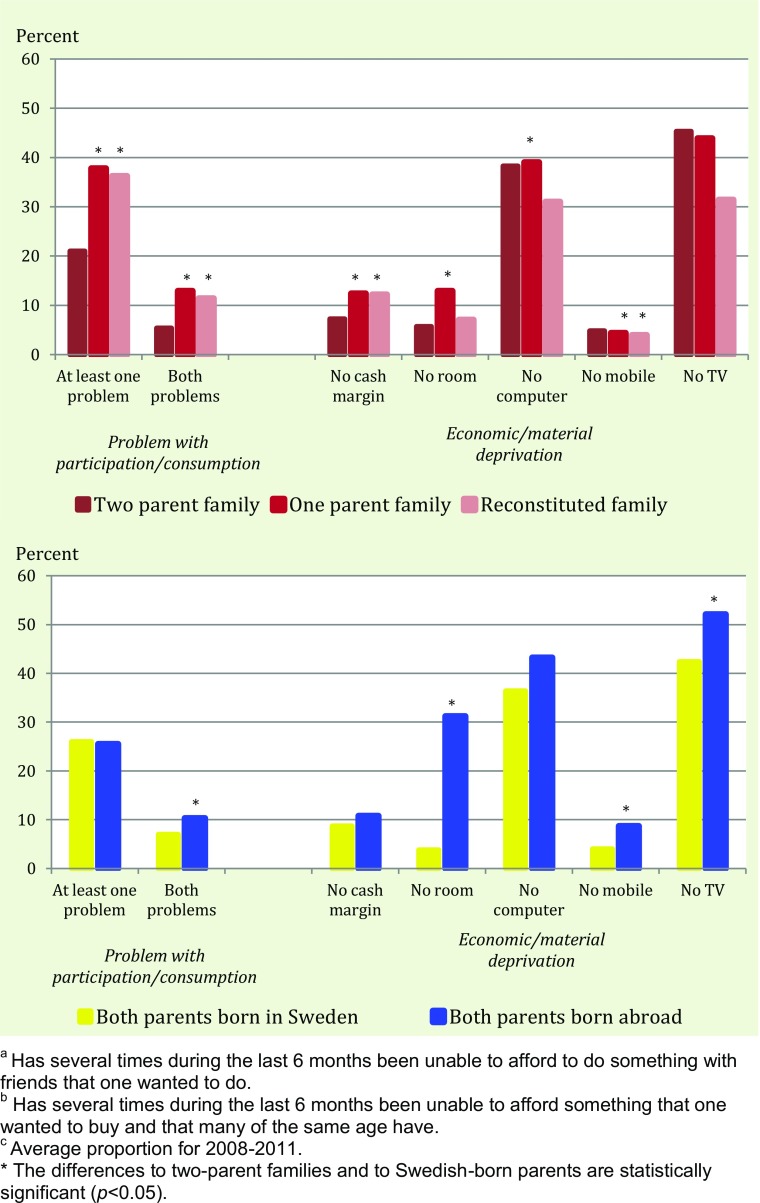
Percentage of 10-18-year-olds in 2008–2011 who have problems with participation^a^ or consumption^b^, or both, and children who lack cash margin or different possessions by family type and parent immigrant origin^c^

As demonstrated in the upper graph of Fig. 6 children who have experienced a parental separation clearly have higher risks for facing problems with consumption or participation, and they also lack a cash margin somewhat more often. It is interesting to note that children in reconstituted families have the same degree of economic problems as children to single parents, despite the fact that the household economy among children with step-parents is almost identical to the one in families with two original parents. This is a strong indication that household income as a liquid resource is not shared with step-children to the same extent as with biological children. When it comes to material standards, there is however no systematic disadvantage for children in reconstituted families.

The lower graph in Fig. 6 describes differences in economic hardship between immigrants’ and the majority’s offspring (excluding children with “mixed” origins). Both consumption and participation problems are relatively equally shared, even though children of immigrants more often have both these problems. Material standards are fairly similar too, with one striking exception: more than 30 % of children of immigrants lack their own room, while this is true for less than 4 % of children of Swedish-born parents. Another difference (not shown) pertains to the regular income from parents, in whatever form – children of immigrants have such money flow less often. In addition, they work less often and therefore have less earned money themselves.

The trends over time in child-reported economic conditions appear to be roughly similar for children in different family types and between immigrants and natives, but our data are somewhat too sparse to draw any firm conclusion about these sub-groups.

## Household Poverty and Child Economic Resources

**Fig. 7:**
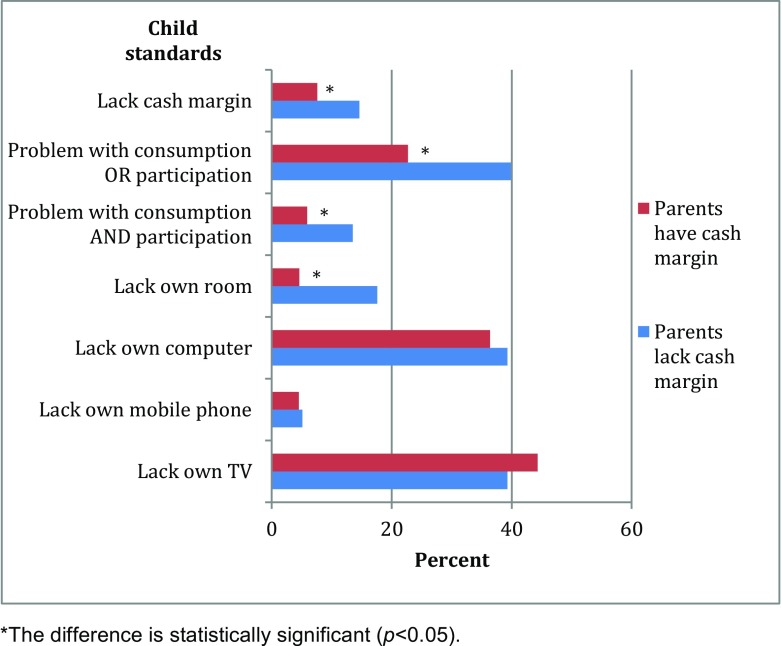
Percentage of 10–18-year-olds in 2008–2011 lacking economic and material resources by parents’ cash margin

We studied the economic situation at the household and child levels, respectively, and it is quite natural to expect a rather strong association between them. Figure 7 tests this by comparing the economic deprivation of parents and children for the period 2008–2011. Indeed, if parents lack cash margin, their children are twice as likely as other children to do so as well (15 % compared to 7.5 %), and the differences are large for the relative child poverty measures. However, we must note that the associations between parent and child deprivation are far from perfect. In fact, a majority of children of economically deprived parents do not report problems, and as many as 85 % have a cash margin in spite of their parents’ economic problems. Again, we see that the material standard is rather high, and the situation for children of poor parents does not differ much in the measured respects from other children’s, with one important exception: the former less often have their own room.

### Parental and Child Poverty as Predictors of Child Outcomes in Other Domains

While economic resources are by themselves an important part of the level of living, much of the research interest in child poverty is motivated by the assumption that poverty has negative consequences in other domains. Traditionally, research on the consequences of poverty focused on long-term outcomes such as educational attainment, nest-leaving and teenage pregnancy (e.g., Duncan and Brooks-Gunn 1997; Mayer 1997), but there is now a growing recognition of the importance of studying outcomes for children while they are children (“wellbeing” in contrast to the traditional “well-becoming”). If poverty impedes children’s chances of making and keeping friends, taking part in social activities, or if it is detrimental to their psychological wellbeing, then this is unquestionably sufficient for child poverty to be regarded as a serious societal problem, no matter whether it has long-term consequences or not (cf. Ben Arieh et al. 2001).

Taking this “here-and-now” perspective on child poverty, an important question is which of household and child-level poverty is the more powerful predictor of negative outcomes in other domains. Main and Bradshaw (2012) point out that the surprisingly weak associations found between poverty and child wellbeing in the previous literature may suggest that parent-reported poverty is not a valid representation of child poverty, and their results reveal that child-reported poverty is indeed more strongly related to wellbeing outcomes. There is as yet only a small but growing literature on the relationship between child-reported poverty and their wellbeing in other domains (Jonsson and Östberg 2004; Olsson 2007; Bradshaw and Main 2012; Main 2014; Gross-Manos 2015), and the findings generally show substantial associations with wellbeing in several domains of life.

In Table 3, we compare poor and non-poor children in terms of their participation in leisure time activities, relations with friends, health, health-related behaviour, safety, and crowded housing (variables described in Appendix 3). We define poverty as (i) household-level poverty, measured as parent-reported lack of cash margin^9^ and (ii) child-level poverty, measured as child-reported lack of own cash margin. Note that it is only the poverty variable that differs, while the child outcome variables are identical in the two models, with all except two being child reported.

**Table 3:** Outcomes for poor and non-poor children (and adjusted differences, non-poor vs. poor) predicted by lack of cash margin at the parental and child levels

	Household poverty			Child poverty		
	Poor	Non-poor	Adj. diff.	Poor	Non-poor	Adj. diff.
Dichotomous child outcome	%	%	%-units	%	%	%-units
Organized sports activity every week	0.54	0.68	0.07	0.62	0.66	0.09
^d^Cannot afford activity	0.06	0.03	−0.02	0.08	0.03	−0.05
^c^Safe in neighbourhood (day)	0.96	0.98	0.01	0.95	0.98	0.02
^c^Safe in neighbourhood (night)	0.74	0.80	0.03	0.65	0.82	0.09
^c^Safe to and from school	0.92	0.95	(0.01)	0.87	0.96	0.05
Vandalization/violence/theft in the neighbourhood^b^	0.23	0.11	−0.08	0.16	0.12	−0.02
Friend at home every week	0.70	0.76	0.04	0.72	0.76	0.07
Visit friend every week	0.78	0.82	0.03	0.78	0.82	0.05
Meet friends in free time every week	0.94	0.96	0.02	0.93	0.96	0.03
No friend in class	0.14	0.10	−0.03	0.10	0.10	(−0.01)
No breakfast every day	0.39	0.23	−0.09	0.24	0.26	−0.04
No lunch every day	0.24	0.16	−0.04	0.16	0.18	(−0.01)
Exercise every week	0.78	0.86	0.05	0.77	0.86	0.06
Smokes every week (age 15+)	0.24	0.13	−0.07	0.21	0.14	−0,05
^c^Alcohol every month (age 15+)	0.32	0.35	(0.02)	0.29	0.35	(−0.00)
Child outcome measured by index or scale^e^	Value	Value	Adj. diff.	Value	Value	Adj. diff.
Psychological problems (scale 0–23)	7.13	6.24	−0.71	7.49	6.16	−1.28
Somatic problems (scale 0–16)	4.83	4.21	−0.45	4.81	4.22	−0.66
Bullied (scale 0–16)	1.72	1.43	−0.22	2.34	1.30	−0.59
Persons/room^b^	1.01	0.84	−0.13	0.93	0.86	−0.04

Children aged 10–18 in 2001–2011

N is approx. 12,600 for items observed 2001–2011; 11,200 for items observed 2002–2011; 2900 for items observed 2009–2011; and 5300 for items observed for ages 15 +

Source: Child-ULF, ULF and ULF/SILC, Statistics Sweden

Differences in parentheses () are not statistically significant, otherwise estimates are significant (*p* < 0.05)

^a^ Adjusted for survey year, sex, age, interaction sex/age, region, parent education, parental health, family type, and parental immigrant status

^b^Information from parents

^c^For the period 2002–2011

^d^For the period 2009–2011

^e^Index mean and std dev: psych (6.4; 3.6), somatic (4.3; 2.9), bullying (1.5; 2.2)

As in previous studies in the area, what we can study here are associations rather than causal effects. Nevertheless, in order to exclude some obvious alternative explanations, we control statistically for a number of background variables, and adjusted differences between poor and non-poor are shown in the rightmost column for each poverty definition in Table 3. These figures can thus not be accounted for by compositional differences between the poor and non-poor in terms of gender, age, residential region, parents’ education, parents’ health, family type, or immigrant background.^10^

Table 3 reveals systematic differences across most domains: poor children, regardless of whether we define the group in terms of their own or their household’s conditions, report less participation in sport activities,^11^ perceive their neighbourhoods as more unsafe at night, live in more crowded homes, have worse health-related behaviours, and report more bullying and worse psychological and somatic health. Even if the sizes of some of these differences are not alarming, they remind us that everyday experiences of poor youth could be burdensome.

The pattern of association between poverty and child outcomes is similar regardless of whether we use parent or child reports on poverty, and associations are of roughly similar size. There are, however, some noteworthy differences such as clearly stronger associations with health problems when using child-reported poverty. This may suggest that the child’s own economy is particularly important for health outcomes, but we cannot rule out the possibility that children with more health problems have a more negative outlook on life and over-report economic problems.^12^ We also see a clearly higher level of reported bullying among poor children when defining poverty by their own economy, but no correspondingly large differences in the friendship variables. Parent-reported poverty is more strongly associated with overcrowding and perceived problems in the neighbourhood, which seems natural because parental economy determines housing choices (but also because these variables are the only parent-reported outcomes).

Our results for child-reported poverty are in line with those from studies from other countries, who also find clear effects on various wellbeing dimensions (Bradshaw and Main 2012; Main 2014; Gross-Manos 2015), but in contrast to Bradshaw and Main (2012) and Main (2014), we find that parent-reported poverty is also rather strongly associated to child wellbeing. This difference can potentially be explained by differences in poverty definitions and in model specification, as we see no theoretical reasons that parental poverty should be more strongly related to child outcomes in Sweden than in England.

Causal effects of economic resources on child outcomes are notoriously difficult to estimate, but previous results suggest that they may not be as severe as is often thought (Dahl and Lochner 2012; Duncan et al. 2011; Mayer 1997). Our aim here is primarily to see whether parent- and child-reported poverty are predictive of problems in different domains, and our analysis does not permit causal conclusions. We believe, however, that for housing- and neighbourhood-related problems, such as overcrowding and safety, a causal effect is plausible as economic resources are fundamental to where and how families live. Outcomes having to do with social relations and participation are also likely to be causally affected by poverty to some extent (cf. Mood and Jonsson 2015, who find a likely causal effect on such outcomes among adults). However, for health and health-related behaviour we find it more difficult to draw conclusions. Maybe children in poorer families exercise less often because it is more difficult to find an attractive form of training on a limited budget – but why do they skip (the free) lunch more often and smoke more? Here, we cannot rule out that economically vulnerable families tend to have other characteristics that we do not capture in our analyses, and which, potentially, are the real causes of behaviour that may affect children’s health.

## Conclusions and Discussion

We use family-based indicators of household poverty as well as child-reported economic resources and problems to unravel child poverty trends in Sweden based on different measures. Our results show that absolute household income poverty (minimum income standard) increased with the recession from 1991 to 1996, and that increasing real incomes reduced poverty among families with children between 2000 and 2006, after which it has remained largely unchanged. While it took around 10 years for poverty rates to return to pre-recession levels following the 1990s macroeconomic collapse, Swedish children did not suffer visibly from the international recession in 2008–2009.

Material deprivation and social assistance rates followed these absolute child poverty trends fairly closely, and these are all counter-cyclical in relation to the macroeconomy. However, increasing income inequality has led to growing rates of relative income poverty since the mid-1990s. A worry is that real income growth, that for a long time offset increasing income inequality for the poorest families, has stalled and therefore the period after 2006, approximately, is characterized by poor children lagging more and more behind those of more economically fortunate backgrounds, without experiencing any improvement in purchasing power.

However, when we instead turn our attention to child poverty as measured by children’s own reports, where we have data for the 2000–2011 period, we could not find any increase in relative poverty that matches the pattern for household poverty. This contradictory pattern may be a consequence of poor parents distributing relatively more of the household income to their children in times of economic duress, as suggested by previous findings (Middleton et al. 1997; Ridge 2002), but it may also be a sign that relative income poverty lacks validity as an indicator of poverty. One mechanism that may slow down negative effects of relative poverty is socioeconomic segregation, which leads poor children to live near, go to school with, and compare themselves predominantly with other poor children.

All in all, we find few negative trends in child poverty as reported by children themselves for the period 2000–2011. This is true for relative poverty, for material possessions, for cash margin, and for income from pocket money and from work for pay. Actually, we find very few trends at all. It appears that, during the period we study, children’s economy was hardly affected by changes in either relative or absolute income poverty trends at the household level, with the exception of an improvement in some indicators of material possessions.

The overall lack of trends in child-reported poverty may raise doubts about the validity of child-reports as measures of economic hardship, but our results clearly show that child-reported poverty is associated with lower quality of life in a variety of domains, and that the pattern is overall similar to the one based on household poverty indicators. We believe that the most reasonable interpretation of our results in this respect is that household- and child-reported poverty mainly capture similar and also partly different dimensions of economic hardship, both being of relevance for children’s everyday lives.

Our results suggest that the trends in both absolute and relative household poverty, and their relation to children’s outcomes, should be followed particularly closely in countries that, like Sweden, have experienced macroeconomic stagnation and growing income inequality. Although we do not register any signs of increasing poverty from our child reports, it may be that negative consequences for children of increasing economic differences emerge only gradually. If real incomes around the median but not the bottom of the income distribution continue to rise, there is a risk that the normal consumption level among children escalates (e.g., in terms of leisure time activities, electronic gadgets or fashion clothing), so that the poorest can no longer keep up with their more fortunate friends and schoolmates.

In Sweden, child poverty measured at the household level is highly dependent on the extent to which a household relies on benefits rather than market incomes. This means that (especially newly arrived) immigrants and single parents are vulnerable groups, something that has been shown repeatedly on one-shot cross-sectional data (e.g., Smeeding et al. 2009; Eurostat 2015). We found that children in these groups experienced similar poverty trends as other children, but also that they are more sensitive to macroeconomic up- and downturns: every third child of a single parent and almost every second of immigrant background fell below the (absolute) poverty line during the last great recession in Sweden, in the mid-1990s. However, tax and family policy are important in determining child poverty rates, so targeting these groups for poverty reduction is possible if the political will could be mustered.

The UN Convention on the Rights of the Child and the associated demands to monitor child wellbeing has been an important force behind putting child poverty on the agenda. Our analyses show the usefulness of studying child poverty using different poverty definitions. There will never be one single poverty measure to capture all of the dimensions that poverty entails and trends may differ according to the measure used. The household poverty measures used here – absolute income poverty, social assistance, relative income poverty, and economic and material deprivation – are informative and relatively simple to follow over time for monitoring purposes. They are, however, not sufficient, but must be complemented with nationally representative data on children’s conditions as reported by children themselves. Such information is crucial for detecting child-relevant trends, indispensable for evaluating the consequences of change in household poverty for child outcomes, and necessary for the further study of the relation between macro- and household economy and child wellbeing.

Collection and analysis of child-reported data on children’s own economic resources is a field that holds great promise for developing our understanding of poverty. It can also make child poverty visible in a way that is both relevant for the target group and easy for policy makers and the general public to comprehend, as it portrays poverty in terms of the lack of tangible everyday resources and opportunities that most people can relate to.

## Appendix A. Data Sources

The following data sources are used in the analyses:

- **The Household Finances Survey (HEK, Statistics Sweden)** is an annual Swedish survey running since 1975. The earliest comparable information in HEK pertains to 1993. However, in some cases, one can use a specially calculated value for 1991, and in these cases the value for 1992 is interpolated as the average of 1991 and 1993. Data is collected by phone interviews covering, for example, household composition, housing, housing costs, childcare, employment, working time, occupation and medical expenses.
  The survey data are matched to register data on, for example, incomes, benefits and taxes. The population consists of Swedish residents 18 years or older during the survey year, excluding people in institutions or in military service. Data is collected for the sampled persons and those in his/her household, and the sample size has varied between 10,000 and 19,000 households. The non-response rate has increased over time, reaching 35 % in 2010.
- **The income and taxation register** (IoT, Statistics Sweden) consists of register data on incomes, taxes and benefits and is available from 1968. In this report, IoT is the source of the income data in HEK, ULF/SILC and LNU.
- **Living Conditions Survey** (ULF/SILC, Statistics Sweden) is a nationally representative survey running since 1975, with an annual sample ranging between 6000 and 8000. ULF collects data on central components of welfare, such as health, economy, work, education, leisure activities and safety. Non-response has been between 20 and 27 % between 1990 and 2008. Several changes in ULF took place 2006–2008, affecting comparability over time. First, the data collection method changed from face-to-face interviews to phone interviews: until 2005 interviews were face-to-face, in 2006 50 %of interviews were face-to-face and 50 % were by phone, and from 2007 on, all interviews have been conducted by phone. Another change was the integration with EU-SILC in 2008, which introduced new questions, and removed or revised several old questions.
- **Living Conditions Survey of Children** (Child-ULF, Statistics Sweden) is a survey covering children aged 10–18 whose parent has been interviewed in ULF/SILC (see above), with an annual sample of around 1000 children. The survey started in 2001, using the same questions as Child-LNU (see below), but it was slightly modified in 2002. As in the ULF/SILC, the data collection method changed in 2005–2007; this affects comparability over time. Child-ULF contains self-reported data on children’s health, school situation, leisure time activities and relations to friends, parents, teachers and other adults. Data can be matched to parental data from ULF/SILC.
- **The Level-of-living Survey** (LNU. Swedish Institute for social research (SOFI), Stockholm University). Panel survey running in 1968, 1974, 1981, 1991, 2000 and 2010 with a sample representative of the adult Swedish population (*N* = approx. 5000–6000 per survey). The face-to-face interviews cover living conditions in a wide range of areas, including work, education, health, economy, leisure time activities, housing and political and civil participation. The age span changed from 15–75 to 18–75 in 1991. From the start, LNU has been a panel study, with replacement for young people and immigrants in order to make each cross-sectional survey representative of the Swedish population (each sample person representing 1/1000 of the population).
- **Child-LNU (**Swedish Institute for social research (SOFI), Stockholm University). A survey running in 2000 and 2010 and covering children (10–18) whose parents are respondents in the LNU (see above). Children report about living conditions such as housing, safety, social relations, school conditions, bullying, wellbeing and economic and material resources. Sample size is approximately 1000 per survey. [35, 59]
- **European Union Statistics on Income and Living Conditions (EU-SILC, Eurostat)** is an annual survey in the EU-countries since 2003. The 2010 survey included the 27 member countries (Belgium, Bulgaria, Cyprus, Denmark, Estonia, Finland, France, Greece, Ireland, Italy, Latvia, Lithuania, Luxembourg, Malta, Netherlands, Poland, Portugal, Romania, Slovakia, Slovenia, Spain, Great Britain, Sweden, Czech Republic, Germany, Hungary and Austria) but also Iceland, Croatia, Norway, Switzerland and Turkey. EU-SILC is representative of the population in each country and collects information on the respondents’ social and economic situation, such as income, deprivation, social exclusion and standard of living. The survey contains both cross-sectional and longitudinal data, and data is collected for individuals but in some cases also for households. The Swedish data is collected by Statistics Sweden.

## Appendix B

Table 4

**Table 4:** Poverty and economic resources: variable definitions

Household economic deprivation	
Indicator	Interview information
Lack cash margin by own means/ Economic deprivation	Cannot raise a sum of money within a week through own assets or savings ^a,b,c^
Lack cash margin entirely/ Economic deprivation	As above, but cannot borrow money either ^a,b^
Economic crisis	Has sometime during the last 12 months had problem making ends meet (pay for food, rent, bills, etc.)
Worried about the private economy	Often or sometimes worried about own or family economy in the upcoming year
Material deprivation	Cannot finance at least three out of nine necessary consumer items or services^d^

^a^ULF 1980–2007. The sum required has been increased over time to compensate, approximately, for inflation: (1980–81: 5000 SEK); (1982–84: 7000); (1985–87: 8000); (1988–89: 9000); (1990–93: 12,000); (1994–95: 13,000); (1996–2003: 14,000); (2005–07: 15,000). In 2006, there was a methods change, from face-to-face to telephone, meaning that estimates for the pre- and post-2006 waves are not comparable

^b^LNU 1968–2010: The sum required has been increased over time to compensate, approximately, for inflation: (1968: 2000 SEK); (1974: 2500); (1981: 5000); (1991: 10,000); (2000: 12,000); (2010: 14,000). For 1991 and 2000, the sum is somewhat lower than in ULF

^c^ULF/SILC 2008–2011: 8000 SEK. At the change from ULF to EU-SILC in 2008, both the sum and the formulation of the question changed, meaning that estimates up to 2007 and from and including 2008 are not comparable

^d^EU-SILC 2005–2010: Necessities: rent, heating, cash margin (1/12 of 60 % or median); eating meat, fish, or equal protein-based meal at least every second day; 1 weeks vacation away; car; washing machine; TV-set; telephone.

### Social Assistance

Social Assistance (SA) is a means-tested benefit given to those who lack sufficient own incomes to have an adequate living standard. The income limits for SA are set for different household types each calendar year by the government, and should reflect reasonable costs for food, clothes, shoes, household items, free-time activities/equipment, health, hygiene, newspaper, TV and telephone. In addition, reasonable costs for housing costs, insurance, electricity, travel and union membership are judged for each household. Costs for special needs, e.g., dental care, glasses, childcare and medicines can also be covered. Our measure indicates whether the household has received SA some time during the year (irrespective of duration or volume) or not.

### Household Income Measures

*Median income* is the income in the middle of the income distribution.

*Disposable income* consists of incomes from work, capital, transfers and benefits, while taxes are subtracted.

*Equivalized disposable income* or *disposable income per consumption unit* adjusts the disposable income by an equivalence scale to reflect the needs as estimated by household size and composition. The equivalence scale used in the Household Finances Survey (HEK) is:

- 1 – one adult
- 1.51 – two adults
- 0.52 – first child 0–18
- 0.42 – later children 0–18
- 0.60 – children over 19 and other adults in the household

*Real income*, reflecting purchasing power, is nominal income that is corrected for inflation (per consumer price index, CPI) in order to be comparable over time.

### Household Income Poverty

**Table:**

Poverty measure	Definition
Low income standard	The household’s equivalized disposable *(absolute poverty)* income is below the threshold for low income standard.^13^
Relative poverty	The household’s equivalized disposable income is below 60 % (or 50 %) of the median income in the country.

### Children’s Economy and Economic Deprivation

**Table:**

Indicator	Information from interview
Income	Works extra (only children 16–18). Gets their child/study allowance. Gets weekly or monthly pocket money.
Material resources (deprivation)	Has the following: own room, own TV, own computer, own mobile telephone
Cash margin	Can get SEK 100 (€9) [SEK 150 in Child-LNU 2010] by tomorrow, e.g., to go to the cinema, if needed.
Participation	Could not afford to join friends for events etc., several times during the last 6 months.
Consumption	Could not afford to buy things that s/he wanted, and that many in her/his age has, several times during the last 6 months.

## Appendix C. Description of Variables in Table 3

Table 3 reports the associations between poverty and 19 different dependent variables, with poverty measured as:

1. Household poverty: Having an income in the lowest quintile and lacking cash margin (access to approx. 1500 Euro in a week).
2. Child poverty, defined as lacking cash margin, i.e., not being able to raise 100 SEK (approx. €10) if needed to the next day. 14

Dependent variables (unless otherwise stated, all are measured 2001–2010):

The child is asked (2002–2010) whether they *feel safe*…

- …in their *neighbourhood daytime*: Yes = 1, No = 0
- …in their *neighbourhood night-time*: Yes = 1, No = 0
- …going *to and from school*: Yes = 1, No = 0

The child is asked (2009–2010): “Is there some leisure activity that many others participate in that you would like to but cannot do?” If the child responds yes, they are asked for the reason (no time, cannot afford, too far, parents do not allow, illness/handicap, no-one to do it with, other). We code *cannot afford activity* as 1 if they give this alternative, otherwise 0.

The child is asked whether they have any close friend in the school class. Those who *lack friends in the school class* are coded 1, others 0.

The child is asked how many days during a normal week that they…

- …have friends at home
- …visit friends in their home
- …spend time with friends in some other place (e.g. outside)
- …participate in some organized sports activity

For each item those who report doing it at least one a week are coded 1, others are coded 0. Using the three variables measuring time with friends, we also construct one variable saying whether one *spends time with friends* at all during a normal week (regardless of whether it is at home, in their home or in some other place).

The child is asked how often, during the last 6 months, that they have…

- …*skipped breakfast*. If at least once a week it is coded as 1, otherwise 0.
- …*skipped lunch*. If at least once a week it is coded as 1, otherwise 0.
- …*exercised*. If at least once a week it is coded as 1, otherwise 0.
- …*smoked*. If at least once a week it is coded as 1, otherwise 0.
- …*drunk alcohol* (2002–2010). If at least once a month it is coded as 1, otherwise 0.

The parent is asked if *vandalization, violence or theft* is common in their neighbourhood. If yes, it is coded 1, otherwise 0.

The parent is asked how many persons there are in the household and how many rooms in the home, and this information is used to calculate *the number of persons per room.*

*Psychological complaints* is an index based on child responses to the following questions: “How well does this statement match?”

- I am almost always in a good mood
- I find it hard to sit still and concentrate
- I am often tense and nervous
- I have enough energy to do things
- I often feel sad or down
- I get angry easily
- I am mostly happy with myself
- I am often grumpy and annoyed

Response options: Matches exactly, matches roughly, matches poorly, or does not match at all. Responses are coded 0 (no problem) to 3 (big problem) and are summed to an index with a scale of 0–24; minimum observed value = 0; maximum observed value = 23; mean = 6.4; standard deviation = 3.6.

*Somatic complaints* is an index based on child responses to the following questions: The past 6 months, how often have you had the following?

- Headache
- Stomach ache
- Problems falling asleep
- Felt stressed

Response options: Every day, several times a week, once a week, a couple of times a month, or more seldom. Responses are coded 0 (no problem) to 4 (big problem) and are summed to an index with a scale of 0–16; minimum observed value = 0; maximum observed value = 16; mean = 4.3; standard deviation = 2.9.

Bullying is an index based on child responses to the following questions How often do you usually experience the following things in school?

- Other students accuse you of things you have not done or things you cannot help
- No one wants to be with you
- Other students show they do not like you somehow, for example by teasing you or whispering or joking about you
- One or more students hit you or hurt you in some way

Response options: Almost every day, at least once a week, at least once a month, once in a while, and never. Responses are coded 0 (no problem) to 4 (big problem) and are summed to an index with a scale of 0–16; minimum observed value = 0; maximum observed value = 16; mean = 1.5; standard deviation = 2.2.

### Control Variables

*Parental health* is the self-rated health of the responding parent coded into three dummy variables (good, bad and in between)

*Immigrant background* is defined as having two parents born abroad (for children in two parent families), or, if one lives with a single parent, as this parent being born abroad.

*Region of residence* is classified into seven areas (so-called h-regions: Stockholm; Gothenburg; Malmö; Other larger cities; Northern remote areas; other Southern, other Northern)

*Parental education* is the responding parent’s highest out of seven levels of education.

*Gender* is coded 0/1, and *age* is coded in years. Their interaction is also included in the models.

*Family type* is based on information from the parent and is coded into three categories: (1) Child lives with both biological/adoptive parents, (2) Child lives primarily in single parent household, (3) Child lives primarily in reconstituted family.

## Footnotes

1 Estimates from survey data always come with some margin of error, but because we focus on trends rather than individual year-to-year changes, and in order to make the graphs readable, we do not report confidence intervals.

2 In fact, the LNU data provide comparable measures of cash margin also for 1968 and 1974, showing much higher levels of deprivation in 1968 in particular (17.5 % lacked all possibilities of raising the sum of money, as compared to 12 % in 2010).

3 To avoid this problem, relative income poverty is sometimes anchored at a starting year, which in our data makes the trends similar to those for absolute income poverty. However, this is theoretically cumbersome as it means using a relative measure without believing in its relative characteristics.

4 In all analyses of Child-ULF and Child-LNU, we use design weights to compensate for the fact that children have different sample probabilities depending on family type. Those with two resident parents, for example, have twice the chance of being included than do those residing with one parent.

5 Children are defined as 0–18-year-olds in Fig. 2, but only youths (10–18) are studied here. However, trends in household income poverty are similar between households with children of different ages.

6 The problem of finding a comprehensive set of indicators for constructing a valid index of material deprivation could be overcome by a thorough inventory of possessions (cf. Gordon et al. 2013; Gross-Manos 2015), but the multipurpose datasets that we use do not have such an inventory. The items included have been chosen because they are central in children’s lives, have significant economic value, and are relatively reliably measured, but they do not form an exhaustive set of possessions.

7 The slight recent decrease in the proportion with their own TV in the Child-ULF data can probably be accounted for by the increasing availability of television via computers.

8 It is a characteristic of the Swedish labour market that virtually no one up to the age of 18 is gainfully employed, although, in theory, children are allowed to leave school after the age of 16.

9 We tested other poverty definitions based on parents’ incomes, which give comparable results in general (but not on all outcomes), thus suggesting that although different definitions capture different individuals, they relate similarly to child outcomes. To make the comparison at least conceptually similar, we compare parents’ lack of cash margin with the same indicator for children.

10 In our models we control for variables that are likely to be exogenous, in other words come prior to poverty, but family type and health may to some extent be endogenous (mediators), which would result in an underestimation of the effect of poverty on outcomes. The poor/non-poor differences are in most cases somewhat higher when not adjusting for these variables. We use OLS regressions for metric/index outcomes and linear probability models (LPM) for dichotomous outcomes, because they, in difference to logistic models, give estimates that are comparable across groups and interpretable in percentage unit terms (Mood 2010).

11 However, only a small group of poor children report not being able to afford some activity that they would like to do, meaning that the economic situation may not be the direct cause for poorer children not to participate. A downward adjustment of aspiration levels among poor children may contribute to the small difference to the non-poor.

12 Although we use the most objective indicator in our set, cash margin, there is still a subjective element in the response, which may be related to some of the outcomes. When we instead use the child indicators of relative poverty (participation and consumption) we get stronger associations for some outcomes, but this may be because of reverse causality or some common underlying factor.
